# Oral Antimicrobial Rinse to Reduce Mycobacterial Culture Contamination among Tuberculosis Suspects in Uganda: A Prospective Study

**DOI:** 10.1371/journal.pone.0038888

**Published:** 2012-07-12

**Authors:** Nelson Kalema, Saskia Den Boon, Adithya Cattamanchi, J. Lucian Davis, Alfred Andama, Winceslaus Katagira, Charles Everett, Nicholas Walter, Patrick Byanyima, Sylvia Kaswabuli, William Worodria, Laurence Huang

**Affiliations:** 1 Makerere University – University of California San Francisco (MU-UCSF) Research Collaboration, Kampala, Uganda; 2 Division of Pulmonary and Critical Care Medicine, San Francisco General Hospital, University of California San Francisco, San Francisco, California, United States of America; 3 College of Health Sciences, Makerere University, Kampala, Uganda; 4 Division of Pulmonary Sciences and Critical Care Medicine, University of Colorado, Denver, Colorado, United States of America; 5 HIV/AIDS Division, San Francisco General Hospital, University of California San Francisco, San Francisco, California, United States of America; University of Cape Town, South Africa

## Abstract

**Rationale:**

Contamination by bacterial or fungal organisms reduces the effectiveness of mycobacterial culture for diagnosis of pulmonary tuberculosis (TB). We evaluated the effect of an anti-microbial and an anti-fungal oral rinse prior to expectoration on culture-contamination rates.

**Methods:**

We enrolled a consecutive random sample of adults with cough for ≥2 weeks and suspected TB admitted to Mulago Hospital (Kampala, Uganda) between October 2008 and June 2009. We randomly assigned patients to oral rinse (60 seconds with chlorhexidine followed by 60 seconds with nystatin) vs. no oral rinse prior to initial sputum collection. Uganda National Tuberculosis Reference Laboratory technicians blinded to the method of sputum collection (with or without oral rinse) processed all sputum specimens for smear microscopy (direct Ziehl-Neelsen) and mycobacterial culture (Lowenstein-Jensen media).

**Results:**

Of 220 patients enrolled, 177 (80%) were HIV-seropositive (median CD4-count 37 cells/uL, IQR 13–171 cells/uL). Baseline characteristics were similar between patients in the oral-rinse (N = 110) and no oral-rinse (N = 110) groups. The proportion of contaminated cultures was significantly lower in the oral-rinse group compared to the no oral-rinse group (4% vs. 15%, risk difference −11%, 95% CI −18 to −3%, p = 0.005). Oral rinse significantly reduced the proportion of contaminated cultures among HIV-infected patients (3% vs. 18%, risk difference −14%, 95% CI −23 to −6%, p = 0.002) but not HIV-uninfected (6% vs. 4%, risk difference 2%, 95% CI −12 to +15%, p = 0.81) patients. However, the proportion of smear-positive specimens (25% vs. 35%, p = 0.10) and culture-positive specimens (48% vs. 56%, p = 0.24) were lower in the oral-rinse compared to the no oral-rinse group, although the differences were not statistically significant.

**Conclusions:**

Oral rinse prior to sputum expectoration is a promising strategy to reduce mycobacterial culture contamination in areas with high HIV prevalence, if strategies can be devised to reduce the adverse impact of oral rinse on smear- and culture-positivity.

## Introduction

Sputum mycobacterial culture services are increasingly offered in low-income countries for the evaluation of patients suspected of pulmonary tuberculosis (TB). This service is in accordance with the latest World Health Organization (WHO) recommendations designed to improve the diagnosis of the more than two million annual smear-negative TB cases and more than 0.5 million multi-drug resistant TB cases [1].

However, contamination of mycobacterial culture media resulting from overgrowth by oro-pharyngeal bacteria and/or fungi is of significant concern. Culture contamination reduces the proportion of interpretable results and diminishes the diagnostic value of culture systems [2]. Studies have reported varying contamination rates of up to 29% in mycobacteria growth indicator tube (MGIT) and 22% in Lowenstein-Jensen (LJ) solid media culture systems [3]–[8].

**Figure 1:**
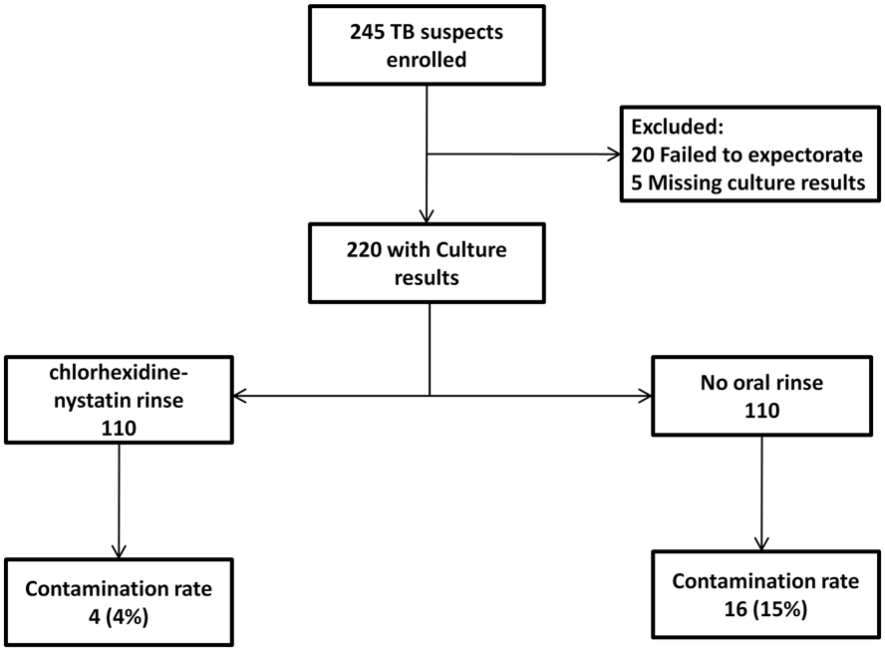
Flow diagram summarizing enrolment and analysis results.

To reduce the risk of sputum culture contamination, WHO recommends strict adherence to standardized laboratory protocols with regard to sputum collection, transportation, and processing [9]. Most TB laboratories process sputum specimens using sodium hydroxide prior to performing mycobacterial culture. However, this also reduces the viability of mycobacteria. Therefore, guidelines recommend varying the concentration of sodium hydroxide to keep contamination rates within the 3–5% target range [10]. Since oral non-mycobacterial flora and *Candida* species are common causes of culture contamination [11], we hypothesized that rinsing the mouth with anti-bacterial (chlorhexidine) and anti-fungal (nystatin) solutions before sputum collection would decrease culture contamination among patients suspected of TB. Thus, our main study objective was to determine the effect of an anti-microbial, anti-fungal oral rinse prior to expectoration on culture-contamination rates. Our secondary objectives were to determine the effect of this oral rinse on smear microscopy and mycobacterial culture.

**Table 1:** Baseline characteristics.

Characteristic	Oral Rinse(N = 110)	No Oral Rinse(N = 110)	p-value
Median age (years)	33 (27–41)	33 (28–39)	0.61
Female	60 (55)	55 (50)	0.50
HIV-seropositive^a^	92 (84)	85 (77)	0.23
Median CD4 count (cells/uL)	45 (14–187)	29 (12–129)	0.45
Co-trimoxazole prophylaxis	43 (47)	48 (56)	0.20
Antiretroviral therapy	18 (20)	19 (22)	0.65
Median oxygen saturation (%)	94 (91–97)	94 (88–97)	0.28
Salivary sputum sample	9 (8)	22 (20)	0.01

Data presented as N (%) or Median (IQR, inter-quartile range),

a n = 177.

## Methods

### Ethics Statement of Approval

The Makerere University Faculty of Medicine Research and Ethics Committee, the Mulago Hospital Institutional Review Board, the University of California, San Francisco Committee on Human Research, and the Uganda National Council for Science and Technology approved the study protocol. All study participants provided written informed consent.

**Table 2:** Effect of chlorhexidine - nystatin oral rinse on culture contamination.

Primary Outcome				
Contamination Rate % (N)	Oral Rinse n = 110	No Oral Rinse n = 110	Risk Difference (95% CI)	p-value
All Patients	4 (4)	15 (16)	−11 (−18, −3)	0.005
HIV-positive^p^	3 (3)^q^	18 (15)^r^	−14 (−23, −6)	0.002
HIV-negative^s^	6 (1)^t^	4 (1)^u^	2 (−12, +15)	0.81

p N = 177, Total HIV positive participants;

q n = 92,

r n = 85.

s N = 43, Total HIV negative participants;

t n = 18,

u n = 25.

### Study Population

We screened consecutive adult patients admitted to Mulago Hospital (Kampala, Uganda) with cough ≥2 weeks but <6 months and with suspected pulmonary TB for study eligibility. We excluded patients who were receiving TB treatment or had a history of TB treatment in the previous two years. Patients unable to expectorate sputum and those individuals whose smear and culture results were unavailable were excluded from the analysis.

### Patient Evaluation and Procedures

Medical officers obtained demographic and clinical information from enrolled participants using a standardized questionnaire. We tested for Human Immunodeficiency virus (HIV) infection in all patients without a confirmed diagnosis of HIV, and measured CD4+ T-lymphocyte counts in those whom we found to be HIV seropositive. On day 1, prior to sputum expectoration, patients either performed a 60-second oral rinse with chlorhexidine (Corsodyl, GlaxoSmithKline) followed in immediate succession by another 60-second rinse with nystatin (Nycostat Oral Suspension, COSMOS Ltd.) or no rinse at all. Study participants were allocated to either group using a list of randomly generated study ID numbers. Under supervision, all patients provided two sputum specimens for Acid Fast Bacilli (AFB) Ziehl-Neelsen (ZN) stain smear microscopy and culture. Laboratory technicians provided standardized instructions on proper sputum expectoration [12], and transported specimens at +4°C to the National Tuberculosis and Leprosy Programme Reference Laboratory (NTRL) within 3 hours of collection.

**Table 3:** Effect of chlorhexidine-nystatin oral rinse on smear and culture positivity.

Secondary Outcomes				
Outcome % (N)	Oral Rinse n = 110	No Oral Rinse n = 110	Risk Difference (95% CI)	p-Value
ZN smear- Positive	25 (27)	35 (38)	−10 (−22, +2)	0.10
LJ culture-positive	48 (51)^y^	56 (53)^x^	−8 (−22, +6)	0.24

y n = 106, & ^x^n = 94; excluded 4 and 16 contaminated cultures respectively.

### Laboratory Procedures

NTRL technicians blinded to sputum collection procedures (with or without oral rinse) evaluated sputum specimens for acid-fast bacilli (AFB) using direct light microscopy (Ziehl-Neelsen staining) and Lowenstein-Jensen (LJ) mycobacterial culture. They decontaminated specimens using the NALC-NaOH (0.5% N-acetyl-L-cysteine, 2% sodium hydroxide, and 1.5% sodium citrate solution) method, concentrated them by centrifugation, and then inoculated the concentrated pellet onto LJ media. NTRL staff checked LJ cultures at least weekly for positive results and considered cultures to be negative if no growth was observed after eight weeks. They assessed the presence of culture contamination using the following criteria: 1) any change in colour or consistency of culture media, 2) development of any liquid or water film over the culture media, and/or 3) presence of non-mycobacterial colonies in culture media (confirmed by visual inspection of colony morphology and/or Ziehl-Neelsen staining).

### Statistical Analysis

We calculated our sample size using an expected reduction of the contamination rate from 18% (observed with conventional methods prior to the study) to a target of 5% using the chlorhexidine-nystatin rinse. Using an alpha of 0.05 and a power of 0.80, we determined that we needed 109 patients in each group. We compared proportions of contaminated cultures, of positive smears, and of positive cultures in each intervention arm using the chi-squared test or the Fischer’s exact test where appropriate. We carried out sub-analyses stratifying results by HIV status. We defined significance in reference to the probability of a two-tailed, type-I error (p-value) less than 0.05. We used STATA 10 (College Station, TX, USA) for analyses.

## Results

### Study Population

Of 245 eligible patients enrolled, 20 (8%) were excluded because they failed to expectorate and 5 (2%) were subsequently excluded because their culture results were missing (Figure 1). Thus, we analyzed data for 220 total patients. There were no significant differences in the baseline characteristics between the oral-rinse and no oral-rinse groups with respect to age (median age 33 yrs vs. 33 yrs, p = 0.61), gender (female 55% vs. 50%, p = 0.50), median oxygen saturation (94% vs. 94%, p = 0.28), HIV seropositivity (84% vs. 77%, p = 0.23), and if HIV seropositive, median CD4-count (45 cells/uL vs. 29 cells/uL, p = 0.45), use of co-trimoxazole prophylaxis (47% vs. 56%, p = 0.20), and use of antiretroviral therapy (20% vs. 22%, p = 0.65) (Table 1). The proportion of patients who provided salivary sputum samples was significantly lower in the oral-rinse group (8% vs. 20%, p = 0.01).

### Impact of Oral Rinse on Culture Contamination

The proportion of contaminated cultures was significantly lower in the oral-rinse group compared to the no oral-rinse group (4% vs. 15%, risk difference −11%, 95% Confidence Interval (CI) −18% to −3%, p = 0.005) (Table 2). Oral rinse significantly reduced the proportion of contaminated cultures among HIV-infected (3% vs. 18%, risk difference −14%, 95% CI −23% to −6%, p = 0.002) but not among HIV-uninfected patients (6% vs. 4%, risk difference 2%, 95% CI −12% to +15%, p = 0.81).

### Impact of Oral Rinse on Smear- and Culture-positivity

The proportion of patients with positive Ziehl-Neelsen smears was lower in the oral-rinse group compared to the no oral-rinse group, although this difference was not statistically significant (25% vs. 35%, risk difference −10%, 95% CI −22% to +2%, p = 0.10) (Table 3). Similarly, when comparing the rinse and no rinse groups, there was a decrease in smear sensitivity (46% vs. 56%, risk difference −10%, 95% CI −28% to +7%, p = 0.25) and an increase in the proportion of scanty smears (22% vs. 13%, risk difference 9%, 95% CI −9% to +28%, p = 0.34). However, neither difference was statistically significant.

Trends for culture positivity were similar to those observed for smear positivity. The proportion of patients with positive cultures (48% vs. 56%, risk difference −8%, 95% CI −22% to +6%, p = 0.24) was lower in the oral-rinse group, and this difference was smaller when contaminated cultures were considered to be negative (46% vs. 48%, risk difference −2%, 95% CI −15% to +11%, p = 0.79).

## Discussion

Culture contamination represents a major threat to the sensitivity of mycobacterial culture, more so in high TB-HIV burden settings, and yet few studies have investigated strategies to lower its occurrence. Recently, one study in HIV-uninfected individuals showed that rinsing with chlorhexidine reduced MGIT culture contamination rates [13]. In this study of HIV-infected and HIV-uninfected individuals, we found that an oral rinse with chlorhexidine and nystatin before sputum expectoration significantly reduced LJ culture contamination rates, having the greatest impact in HIV-infected patients for whom contamination rates without oral rinse were as high as 18%. Contamination rates among HIV-uninfected participants were low and around 5%, irrespective of the rinse, a result that should be interpreted with caution because the numbers in this group were very small. However, it is possible that the organisms that contribute to contamination either are absent or are present in such low numbers as not to cause any significant contamination. We suspect that such high contamination rates in HIV-infected patients is due to the higher prevalence and density of opportunistic bacterial and fungal oral flora in this population and published data has already implicated Candida species and other common oral bacteria as the potential sources of contamination in this group [14]–[15]. Knowing the diversity and density of these oral microbiota would potentially inform a focused approach to targeting these organisms, and therefore reduce contamination rates. Since HIV-seropositive patients may more likely be AFB smear-negative, culture confirmation of TB is especially important in this group, because failure to do so may lead to missed diagnosis or at least delays in diagnosis and initiation of anti-TB treatment, resulting in high morbidity and mortality and continued disease transmission [16].

An unexpected finding of the study was that chlorhexidine-nystatin oral rinsing showed a trend towards reducing the overall proportion of positive sputum smears and cultures, as well as smear sensitivity. This finding does not have a clear explanation. The reduction in Ziehl-Neelsen smear sensitivity and proportion of positive smears was especially surprising because acid-fast bacilli should still be identified, even when they are non-viable. The reduction in the proportion of positive cultures on the other hand could be explained by a possible but undocumented anti-mycobacterial effect of the chlorhexidine and nystatin rinse. Perhaps some of the rinse is retained within the mouth, such that these agents are expectorated with the sputum specimen in high enough concentrations to inhibit mycobacterial growth. However, whereas chlorhexidine oral rinse has been used and shown to have antibacterial properties against a wide range of oro-microbial bacteria, evidence has shown that mycobacteria are highly resistant to it [17] and recoverable [18], and although nystatin has activity against fungi and yeasts, we are unaware of any studies documenting an anti-mycobacterial effect. An alternative to rinsing with anti-microbial solutions could be a water rinse before sputum collection. One non-randomized study also from Mulago Hospital but conducted by a different research group showed that rinsing with sterile water before sputum expectoration reduced the median monthly culture contamination rate from 22% to 7% [4]. However, no information was provided on the HIV status of the participants or on the proportions of positive smears and cultures, which limits the conclusions that can be definitively drawn. These findings require further investigation and a randomized study comparing water rinse to chlorhexidine and nystatin rinse should be done.

This study has some limitations. First, although we have strong evidence of the negative impact of oral rinse on smear and culture positivity, we did not have a sufficient sample size to estimate precisely its effects on smear and culture sensitivity, more so for the HIV-uninfected population. A larger study is needed to explore this unexpected finding. Second, we only evaluated the combined effect of chlorhexidine and nystatin and are unable to distinguish between the individual contributions of these agents on both culture contamination and on smear- and culture-positivity. It is possible that rinsing with a single solution may be as effective as rinsing with both solutions. Finally, we evaluated the effect of oral rinse on contamination of LJ cultures because MGIT cultures for which strategies to reduce contamination are even more important [3] [6] [13] were not yet available for patient care at Mulago Hospital at the time of the study.

Culture is an important tool for evaluating TB suspects, with special utility for identifying smear-negative TB cases and for testing second-line drug-susceptibility to guide therapy in patients with multi-drug-resistant TB. However, contamination is a major barrier to its efficacy. The use of an antibacterial-antifungal oral rinse prior to sputum expectoration to reduce mycobacterial culture contamination is a promising strategy worth considering, especially in areas with a high HIV prevalence. Further studies are needed to characterize the nature of contaminants in this population and to evaluate the potential adverse impact of each oral rinse in varying concentrations on smear- and culture-positivity.
